# Laparoscopic cholecystectomy in situs inversus totalis: a case report

**DOI:** 10.1186/1471-2482-5-5

**Published:** 2005-03-17

**Authors:** Damian McKay, Geoffrey Blake

**Affiliations:** 1 The Department of Surgery, Daisy Hill Hospital, 5 Hospital Road, Newry, Co Down, BT35 8DR Northern Ireland

## Abstract

**Background:**

Laparoscopic cholecystectomy is one of the commonest surgical procedures carried out in the world today. Occasionally patients present with undiagnosed situs inversus and acute cholecystitis. We discuss one such case and outline how the diagnosis was made and the pitfalls encountered during surgery and how they were overcome.

**Case presentation:**

A 32 year old female presented to our department with epigastric pain radiating through to the back. A diagnosis of acute cholecystitis in a patient with situs inversus totalis was made following clinical examination and radiological investigation. Laparoscopic cholecystectomy was subsequently performed and the patient made an uneventful recovery.

**Conclusion:**

Situs inversus presenting with acute cholecystitis is very rare. The surgeon must appreciate that care should be taken to set up the operating theatre in the mirror image of the normal set-up for cholecystectomy, and that right handed surgeons must modify their technique to adapt to the mirror image anatomy.

## Background

In 1600 the first known case of situs inversus in humans was reported by Fabricius [1]. The incidence is thought to be in the region of 1:5000 to 1:20000 [2]. The condition may affect the thoracic organs, abdominal organs or both. It is associated with a number of other conditions such as Kartagener's (bronchiectasis, sinusitis, situs inversus) and cardiac anomalies. There is no current evidence that situs inversus predisposes to cholelithiasis [3].

Since Mouret first performed it in 1987, laparoscopic cholecystectomy has become the standard operative procedure for gallbladder disease. It is associated with reduced hospital stay, fewer respiratory complications, less pain and a faster return to work.

## Case presentation

A thirty two year old female was admitted with a three hour history of epigastric pain radiating into her back in keeping with biliary colic. She had vomited a number of times. In the previous week she had two episodes of a similar nature.

On examination there was no jaundice or pyrexia. The apex beat was in the right fifth intercostal space, midclavicular line. She had epigastric tenderness but was not tender in the right or left upper quadrants. Her white cell count and amylase level was normal but her C-reactive protein level (CRP) was elevated at 290 mg/L. An electrocardiograph showed right axis deviation and right ventricular hypertrophy, in keeping with dextrocardia.

An ultrasound scan of the upper abdomen identified the gallbladder, which contained stones, in the left upper quadrant. The spleen was visualised in the right upper quadrant. There was no evidence of common bile duct or intrahepatic duct dilatation. Chest X-Ray confirmed the clinical and electrocardiograph diagnosis of dextrocardia.

The diagnosis of acute cholecystitis and situs inversus was made. The patient settled clinically over two to three days and was discharged home to be admitted electively for laparoscopic cholecystectomy.

In order to conduct the laparoscopic cholecystectomy all theatre equipment including diathermy, monitors and CO_2 _insufflator were positioned in the mirror image of their normal position. The surgical team also changed sides with the primary surgeon and first assistant on the patients right and the second assistant on the left. The ports were inserted in the usual way but on the left side. At laparoscopy the entirety of the abdominal contents were indeed reversed.

The main difficulty encountered was that the primary surgeon, who was right handed, would have had to cross hands to retract on Hartmann's pouch while dissecting Calot's triangle. We overcame this difficulty by allowing the first assistant to retract on Hartmann's pouch, while the primary surgeon dissected Calot's triangle using his right hand via the epigastric port without hindrance. The common bile duct and cystic duct were identified, as was the cystic artery, which lay anterior to the cystic duct. The surgery proceeded without incident and the patient recovered and was discharged the next day.

## Conclusion

In this case the patient presented with epigastric pain only and had no definite left upper quadrant pain. It has been noted in 30% of previous reported cases of acute cholecystitis in patients with situs inversus that the pain was felt in the epigastrium alone and in 10% the pain was localised to the right upper quadrant [4]. The proposed explanation for this is that the central nervous system may not share in the general transposition.

Previous reports have confirmed that situs inversus is not a contraindication for laparoscopic cholecystectomy [1-3,5,6]. The procedure is, however, more difficult and care and time must be taken to re-arrange the equipment set-up in theatre, and to recognise the mirror-image anatomy which can cause difficulties with orientation. At least two thirds of surgeons are right handed. It is necessary for these surgeons, and their assistants, to modify their usual surgical technique to comfortably and safely carry out the procedure. Rather than the clumsy crossing of hands to retract on Hartmann's pouch for dissection of Calot's triangle, we suggest that retraction on Hartmann's pouch may be carried out by the assistant, thus allowing the surgeon to operate in a more ergodynamic fashion.

### Learning points

1. Detailed clinical examination is important in diagnosing previously unknown situs inversus.

2. Patients with gallabladder disease and situs inversus may have pain in the right upper quadrant, epigastrium or left upper quadrant.

3. Theatre equipment must be moved to the mirror image of their normal positions before surgery.

4. The surgeon must recognise the mirror image anatomy and modify his or her technique appropriately.

## Abbreviations

CRP- C reactive protein.

CO_2_- Carbon dioxide.

## Competing interests

The author(s) declare that they have no competing interests.

## Pre-publication history

The pre-publication history for this paper can be accessed here:


